# Condolence letter for Dr Mwelecele Ntuli Malecela (1963–2022)

**DOI:** 10.1186/s40249-022-00950-8

**Published:** 2022-03-07

**Authors:** Xiao-Nong Zhou

**Affiliations:** National Institute of Parasitic Diseases at China CDC, WHO Collaborating Center for Tropical Diseases, Shanghai, People’s Republic of China



With a deep sense of sadness and sorrow, I learned that Dr Mwelecele Ntuli Malecela, Director of WHO’s Department of Control of Neglected Tropical Diseases, passed away on 10 February 2022 after a prolonged illness at the age of 58. I would like to extend my deepest condolences for the sad loss of Dr Mwele Malecela, a well-known scientist and a long-time friend. She was among the most well-known and respected female researchers, scientists and public health leaders in Africa.

Before her appointment in December 2018 as Director at WHO headquarters in Geneva, Switzerland, she was Director in the Office of the Regional Director, WHO Regional Office for Africa in Brazzaville, Congo, where she was responsible for providing policy, managerial and diplomatic advice to Dr Matshidiso Moeti.

Before joining WHO in April 2017, Dr Malecela served as the Director-General of the National Institute for Medical Research, United Republic of Tanzania and as founding Director of the Tanzania Lymphatic Filariasis Elimination Programme.

Lymphatic filariasis was her main academic interest, starting as a junior scientist focusing on immuno-epidemiology of filarial infections.

Mwelecele Ntuli Malecela was born on 26 March 1963 in Dar es Salaam, daughter of former Tanzanian Prime Minister and Permanent Representative to the United Nations, John Malecela. Mwele, as she was widely known, attended Weruweru Girls Secondary School and later enrolled at the University of Dar-es-Salaam, Tanzania, from where she graduated in Zoology. She completed her PhD in parasitology at the London School of Hygiene and Tropical Medicine.

Her short time as Director of the Department of the Control of Neglected Tropical Diseases culminated most notably in developing the new road map for 2021–2030. The blueprint "Ending the neglect to attain the Sustainable Development Goals: a road map for neglected tropical diseases 2021–2030" was launched in January 2021.

Throughout her life, she advocated for the empowerment of women, gender equality, and the welfare of women and girls. She also was a champion in encouraging and inspiring youth to study science and to serve the cause of global health as viable and vital avenues for realising their talents.

Dr Malecela’s contribution to China-Africa cooperation on schistosomiasis control and elimination was incalculable. Back in 2015, I had the good fortune to meet Dr Malecela in Beijing when she served as the Director-General of the National Institute for Medical Research, United Republic of Tanzania. Owing to her indispensable support, we signed the agreement to establish the Institutional-Based Network of China-Africa Cooperation on Schistosomiasis Elimination. After she joined WHO in 2017 as Director of the Department of the Control of Neglected Tropical Diseases, I was beyond honored to visit her office in Geneva and had an official and fruitful discussion about deepening exchange and collaboration between China and WHO/NTD, followed by a series of virtual meetings after I came back to China. Dr Malecela provided her vision, ideas, and insights that benefited and inspired us in the long term.

She continuously advocated for global fight against the neglected tropical diseases, which will always be remembered and cherished by us.

Her death will be felt deeply and personally by many across the globe, and her inspiration, enthusiasm, and unstinting engagement will continue to guide all those who knew her.

Our sincere condolences are with Mwele’s family and close relatives, as colleagues and friends in the global health community mourn her loss.

## Data Availability

Not applicable.

